# Reciprocal Associations Between Science Efficacy, STEM Identity and Scientist Career Interest Among Adolescent Girls within the Context of Informal Science Learning

**DOI:** 10.1007/s10964-023-01868-6

**Published:** 2023-10-11

**Authors:** Mengya Zhao, Emine Ozturk, Fidelia Law, Angelina Joy, Ashley R. Deutsch, Christina S. Marlow, Channing J. Mathews, Luke McGuire, Adam J. Hoffman, Frances Balkwill, Karen P. Burns, Laurence Butler, Marc Drews, Grace Fields, Hannah Smith, Mark Winterbottom, Kelly Lynn Mulvey, Adam Hartstone-Rose, Adam Rutland

**Affiliations:** 1 University of Exeter, Exeter, UK; 2 North Carolina State University, Raleigh, NC USA; 3 Purdue University, West Lafayette, IN USA; 4 University of Virginia, Charlotteville, VA USA; 5 Cornell University, Ithaca, NY USA; 6 Centre of the Cell, Queen Mary University of London, London, UK; 7 Virginia Aquarium & Marine Science Center, Virginia Beach, VA USA; 8 Thinktank Science Museum, Birmingham, UK; 9 EdVenture, Columbia, SC USA; 10 School District Five of Lexington and Richland Counties, Irmo, SC USA; 11 The Florence Nightingale Museum, London, UK; 12 University of Cambridge, Cambridge, UK

**Keywords:** Science efficacy, STEM identity, Career interest, Informal science learning, Adolescents, Girls

## Abstract

Limited research has explored the longitudinal pathway to youth career interests via identity and efficacy together. This study examined the longitudinal associations between science efficacy, STEM (science, technology, engineering and math) identity, and scientist career interest among girls who are historically considered as an underrepresented group among scientists. The sample included 308 girls (M _age_ = 15.22, SD _age_ = 1.66; 42.8% White) from six STEM youth programs, each at a different informal science learning site within the U.K. and the U.S. Longitudinal structural equation modelling demonstrated that science efficacy consistently predicted STEM identity and scientist career interest, and similarly, STEM identity consistently predicted science efficacy over a two-year period. Scientist career interest at 12 months predicted science efficacy at 24 months. The coefficients of efficacy predicting STEM identity and scientist career interest were significantly larger compared to STEM identity and scientist career interest in predicting science efficacy from 12 months to 24 months. Further mediation analysis supported a significant pathway from STEM identity at 3 months to scientist career interest at 24 months via 12-month science efficacy. The findings highlight that science efficacy and STEM identity for girls relate to their scientist career interest and these longitudinal associations are reciprocal. This study suggests that science efficacy and STEM identity mutually influence each other, and enhancing science efficacy and STEM identity is key to promoting adolescents’ interest in being a scientist.

## Introduction

Women are persistently underrepresented in the science, technology, engineering and math (STEM) workforce (Mulvey et al., 2022), and girls’ STEM career interests decline during high school (Sadler et al., 2012). A potential barrier to girls’ pursuit of STEM careers, and science careers in particular, may be a lack of STEM identity, as high school girls link math, chemistry, and physics with masculinity (Makarova et al., 2019). Research is needed to examine the potential pathways of girls’ scientist career interests, and which factors are critical in inhibiting the decline in scientist career interests among girls during adolescence. This study examines the longitudinal associations between science efficacy, STEM identity and scientist career interest among girls who participate in youth programs within informal science learning sites (e.g., science museums/centers, zoos and aquariums) in the United Kingdom (U.K.) and United States (U.S.).

### How Do Science Efficacy and STEM Identity Link with Scientist Career Interest?

Science efficacy refers to an individual’s belief in their ability to complete science tasks and to solve science problems (Ackert et al., 2021), and scientist career interest is defined as an individual’s interest in choosing a career as a scientist in the future (Luo et al., 2021). According to Social Cognitive Career Theory (Lent et al., 2000), self-efficacy is a reliable predictor of an individual’s career interest. Social Cognitive Career Theory (Lent et al., 2000) highlights that an individual’s belief in their ability to excel in a relevant career area (e.g., science efficacy) is shaped by learning experiences (Lent & Brown, 2019). As such, it is expected that girls in the STEM youth programs (a type of learning experience) with a higher level of science efficacy may express a higher level of scientist career interest in alignment with the Social Cognitive Career Theory’s propositions. Previous research supports this proposition, showing that female high school students who are higher in science efficacy are more likely to choose a STEM major in college compared to those lower in science efficacy (Sahin et al., 2017).

However, science efficacy may not be associated with scientist career interest. For example, research found that although boys’ math efficacy in middle school was linked with science career interests, this association was not observed in girls (Huang et al., 2019). Research looking at the association between science efficacy and persistence in science career intentions in a group of undergraduates, who participated in summer research experience programs, also found that neither baseline science efficacy or science efficacy assessed around one month after joining the summer program was correlated to career persistence intentions (Hernandez et al., 2018). These findings suggest that for girls who are underrepresented in STEM careers, their lower levels of STEM career interests may not simply be due to their perceptions of themselves as lacking in the ability or competence to do STEM activities (i.e., lower STEM efficacy). There may be other individual or contextual factors that are important when explaining the development of STEM career interests among youth, especially among girls. Science efficacy may not always play a role in the development of scientist career interest, especially among girls, given that girls are historically under-represented in STEM fields (Huang et al., 2019).

Social identity may be one of the factors which is critical for girls’ STEM career interests (Kim et al., 2018). For example, informal science learning sites that are inclusive and engender a sense of belonging among underrepresented youth can support young people in preparing for their careers (Zhao et al., 2023). Further, research has shown that STEM identity is associated with STEM career motivation and STEM identity mediates the connection between gender stereotyping and STEM career motivation among female undergraduates (Starr, 2018). Together, these studies suggest that STEM identity may be key to fueling scientist career interest among adolescent girls.

STEM identity is operationalized as the perceived identity compatibility between the self (i.e., personal identity) and STEM. Social Identity Theory contends that a person’s social identity can be a key motivating factor for their behavior because individuals who develop a sense of personal belonging within a social category are more likely to act positively towards it (Tajfel & Turner, 1986). Therefore, a stronger STEM identity among adolescent girls should be associated with a sense of belonging to and feeling of acceptance by the STEM community (Kim et al., 2018). Empirical evidence supports an association between belonging and perceived career preparation in youth at informal science learning sites (Zhao et al., 2023). Research suggests that STEM identity is linked to STEM career intentions in undergraduates, and STEM identity is associated with early informal science learning experiences, such as attending science camps (Dou et al., 2019). Similar findings have been observed in middle school girls, with their perceptions in relation to science associated with STEM-related career interests (Kang et al., 2019). Further, a 10-week longitudinal study among undergraduate students found that the change in STEM identity at the beginning and end of the term was positively associated with the change in STEM career aspiration at the beginning and end of the term (Starr et al., 2020).

### Science Efficacy, STEM Identity and Scientist Career Interest are Mutually Associated with Each Other

While research has documented the association between science efficacy and STEM identity with scientist career interest, as discussed, the direction of the association may be bidirectional rather than only efficacy and identity predicting career interest. For example, using a cross-lagged panel design, a study found that career interests and efficacy were reciprocally related to each other across three-time points in college students (Nauta et al., 2002). Evidence from an experimental study involving undergraduate students revealed that higher interest in a certain occupation (i.e., career interest) predicted a higher confidence in the ability to do that occupation (i.e., efficacy) (Bonitz et al., 2010). There is limited research on the bidirectional association between STEM identity and scientist career interest, but burgeoning research into social identity and academic performance supports the bidirectional associations (Bonitto, 2020; Gulemetova et al., 2022).

Similarly, science efficacy and STEM identity may be mutually related. Based on the Persistence Framework (Graham et al., 2013), STEM identity and efficacy are key to being persistent in STEM, and the underlying psychological process may be a cycle, with youth developing higher science efficacy which enhances their STEM identity, and then this STEM identity further increases their science efficacy. However, limited research has used longitudinal designs with repeated measures to explore the bidirectional relationships between science efficacy and STEM identity among youth. Very little research evidence supports the bidirectional associations between efficacy and identity proposed by the persistence framework (Graham et al., 2013). For example, a study explored the longitudinal relationships between science efficacy and science identity among undergraduate students during their study over eighteen months (Robnett et al., 2015). They found that science identity at baseline predicted science efficacy six months later but efficacy at baseline did not predict science identity six months later. Likewise, from six months to eighteen months only science identity predicted science efficacy and science efficacy no longer predicted science identity. The same study also found that research experiences at baseline predicted science identity after 18 months, and this association was mediated by science efficacy around six months after baseline. This work has not included career-related variables, which may also be important. Thus, the current study will extend this work to investigate the role of STEM identity and science efficacy in scientist career interest.

### The Importance of Participation in STEM Youth Programs

Recently research has investigated the benefits of informal science learning, and a literature has accumulated showing that participation in STEM youth programs, where adolescents and young people work at informal science learning sites as educators interacting with visitors, plays a key role in the development of science efficacy (Hoffman et al., 2021), STEM identity (Hughes et al., 2013), positive STEM developmental trajectories (Joy et al., 2023) and career preparation (Zhao et al., 2023). Adolescents who participated in a science research program at a natural history museum reported that they developed research skills through the program and maintained their science interests outside of the program (Habig & Gupta, 2021). This research program also facilitated adolescents’ STEM persistence, such as choosing a STEM career (Habig et al., 2020). Stronger evidence from a meta-analysis reviewing the literature from 2009–2015 supports the importance of youth STEM programs for STEM interests and shows that out-of-school programs have a positive effect on adolescents’ STEM interests (Young et al., 2017). Collectively, research highlights the importance of participation in STEM youth programs for adolescents’ positive STEM trajectory development (e.g., interest, identity) and STEM persistence (e.g., major choice and career choice) in general.

Research also documents the importance of participation in STEM youth programs among girls in particular. For example, a museum-based science program was found to enhance girls’ STEM interest, motivation, and persistence (Adams et al., 2014). Beyond that, research highlights the long-term impacts of informal STEM programs on awareness and understanding of science, science identity, and shaping future education and careers among girls (Dierking, 2014). Research shows that participating in a STEM camp within the informal science learning context improves girls’ STEM identities (Hughes et al., 2013).

The Persistence Framework (Graham et al., 2013) has identified that membership in STEM learning communities (e.g., youth programs at informal science sites) can influence individuals’ science efficacy and identity, which are key to STEM persistence (e.g., enrolling in STEM courses and choosing a STEM career). As such, participation in STEM youth programs at informal science learning sites may influence girls’ science efficacy and STEM identity, which would, in turn, could influence girls’ scientist career interest.

## Current Study

**Fig. 1:**
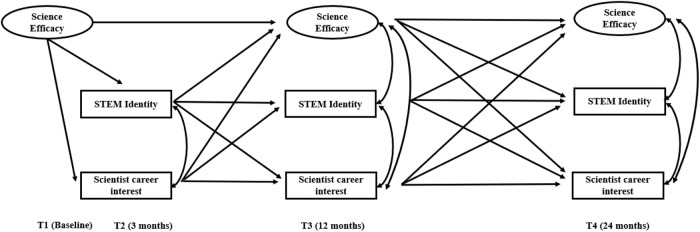
Hypothesized Model

There is a need to understand the underlying psychological process behind the emergence and maintenance of science career interest among girls in order to increase girls’ representation in STEM. This study aims to investigate the longitudinal associations between science efficacy, STEM identity and scientist career interest and explore the direction of the longitudinal associations between these variables among girls who participate in STEM youth programs at informal science learning sites. The aim is to identify key factors which may inhibit the decline in science career interests commonly found among girls during adolescence. This study addresses two research questions. First, integrating the Social Cognitive Career Theory and Social Identity Theory, the study examines whether science efficacy and STEM identity longitudinally relate to girls’ scientist career interest and explores potential longitudinal pathways involving science efficacy and STEM identity to scientist career interest. Second, drawing on the Persistence Framework, the study investigates if the association between science efficacy and STEM identity is bidirectional. The current research used a longitudinal design to examine the factors related to scientist career interest in girls who joined STEM youth programs at informal science learning sites (hypothesized model; see Fig. 1). It is expected that science efficacy and STEM identity will positively predict scientist career interest (Hypothesis 1). It is anticipated that scientist career interest will predict science efficacy and STEM identity (Hypothesis 2). Third, it is expected that a mutual association between science efficacy and STEM identity will be observed (Hypothesis 3). Last, given the potential bidirectional associations between science efficacy, STEM identity and scientist career interest, there is no specific hypothesis related to the longitudinal pathway from science efficacy and STEM identity to scientist career interest.

## Methods

### Participants

The sample includes 308 girls who took part in a longitudinal survey from STEM youth programs at informal science learning sites in the U.K. (43.5%) and the U.S. (56.5%). Participants were aged from 10 to 21 years old when they first joined the youth program (*M*_*age*_ = 15.22, *SD*_*age*_ = 1.66; 15 participants did not report their age). The participants in the study were racially and ethnically diverse: 5.5% of the total sample was White British, 20.1% South Asian British, 5.2% Black British, 3.6% Dual Heritage British, 37.3% White/European American, 2.6% Asian American, 8.1% Black American, 1.9% Hispanic/Latino/e/a/x, 4.2% Mixed-Race/Bi-racial American. Seven participants declined to report their race or ethnicity.

Participants were recruited from six informal science learning sites (three from the US and three from the UK). The UK sites included a biomedical and cell biology science education center (31.5%), a science museum (9.5%) and a medical heritage museum (2.9%). The biomedical and cell biology science education center and the medical heritage museum are located in the same city, but the science museum is located in another city. The US sites included an aquarium (36.1%), a zoo (13.8%), and a children’s museum (6.9%). Similarly, the zoo and the children’s museum are located in the same city, and the aquarium is located in another city. The main task for youth participating in the STEM youth programs is to interact with visitors. Youth work as youth educators at the site to facilitate visitors’ learning, such as, demonstrating interactive exhibitions/interacting with animals and sharing relevant STEM knowledge. The youth programs encourage youth to design exhibitions/information cards for the site. Youth can attend workshops related to their career development, such as networking and career mentorship. Although the content or focus for the youth programs is different across sites, all programs involve youth interacting with visitors, creating science content for their sites, and career development activities. The STEM youth programs expect youth to develop their STEM-relevant knowledge and skills as well as other critical soft skills (e.g., communication, planning). The programs recruit youth from a wide range of ages. Youth in each program were required to participate for at least one full year, but most continued to stay involved for multiple years.

### Procedure

Ethical approval was obtained at the beginning from two Universities in one application (Ethics approval number is 21017). A joint research team from the University of Exeter and North Carolina State University recruited the participants for the longitudinal study. Parents were informed and youth assents were obtained when the participants joined their respective youth programs. The research team sent a Qualtrics survey via email to the participants and, for those completing the survey, low-value electronic gift cards were sent out as an expression of gratitude.

The researchers coordinated data collection with practitioners at the site to recruit participants at the beginning of their participation in the program. Once participants were enrolled in the study, they were sent the survey links at specific time intervals based on their initial enrolment data. The longitudinal data were collected at four different time points: the first time point (T1) was a baseline assessment, and the measures were sent out at the beginning of the program when the participants were in their induction session (it took one month for all participants to complete the first survey); the second time point (T2) was around 3 months after the start of the program; the third time point (T3) was 12 months after joining the program; the fourth time point (T4) was 24 months. The measures in the T1 survey and T2 survey were different: the survey at T2 included a smaller bank of measures related to STEM identity, career interests and feelings of belonging, and this brief survey aimed to reduce participants’ burden. The measures in T3 and T4 surveys were the same and were the combined measures from T1 and T2 surveys. This decision to combine the measures into a big survey was made to reduce the missingness.

### Measures

#### Science Efficacy

Five items assessing youth’s beliefs regarding their science efficacy were adapted from previous research (Bandura et al., 2001). Participants were asked to respond to the items on a seven-point scale. One example question reads, “how good would you be at learning something new in science?” (1 = *not at all good*; 7= *very good*). Science efficacy was assessed at T1, T3, and T4 (*α*_T1_ = 0.90; *α*_T3_ = 0.90; *α*_T4_ = 0.88), but not T2. Confirmatory factor analysis further supported the factor structure of science efficacy at T1, T3 and T4, and science efficacy was composed of the same measure over time (science efficacy was not assessed at T2; model fits see Table S1 and factor loadings see Table S2 in Supplementary materials).

#### STEM Identity

Participants were asked to look at seven pictures, with each picture including a set of two circles representing “You” and “STEM” respectively. Each pair of circles had differing degrees of overlap between the two circles. Participants were asked to select one of the seven sets of overlapping circles that best represents the compatibility between their personal and STEM identities. Previous research has provided detailed validity and reliability evidence for this measure regarding assessing STEM identity (McDonald et al., 2019). STEM identity was assessed at T2, T3, and T4, but not T1.

#### Scientist Career Interest

Participants were asked “Do you think that you will be a scientist when you choose a career?” They rated their scientist career interest on a 7-point scale (from 1 “absolutely not” to 7 “absolutely”). This item was assessed at T2, T3, and T4, but not T1.

#### Control Variables

Age and ethnicity were controlled in the model. Differences were not expected between the UK and US. Nonetheless country was also controlled in the model.

All items used in the current study can be found in the supplementary material.

### Data Analysis

SPSS 25.0 was used for internal consistency, descriptive analyses, and missing data description. MPlus 8.4 statistical software was used for longitudinal structural equation modeling (SEM). First, missing data analysis was conducted to understand the percentage, and missing pattern, of the missingness. The percentage of missingness for each variable (Table S3 in supplementary materials) was evaluated by frequency analysis. Based on the frequency analysis, the data was not normally distributed. The mean percentage of missingness at T1, T2, T3 and T4 was 9.7%, 46.8%, 48.7% and 50.8%, respectively. Furthermore, only around 22% of participants (*n* = 68) answered all items across four time points. There was substantial missing data in the study from T1 to T4. Therefore, the completed case analysis (*n* = 68) is likely impacted by selection bias and small sample size. Full information maximum likelihood (FIML) was used to address missingness for the next step of the analysis (Enders & Bandalos, 2001).

Given that the measures in the study used ordinal data, the weighted least squares mean and variance adjusted estimation (WLSMV) in Mplus is recommended (Muthén, 1984). However, the default setting of WLSMV in Mplus to deal with missingness is pairwise deletion, which is not suitable for 50% missingness. Thus, robust maximum likelihood (MLR) estimation and full information maximum likelihood (FIML) were used for the next step of data analysis, which allow for missing data management (Chen et al., 2020) and which account for the non-normal distribution (Little, 2013).

Confirmatory factor analysis (CFA) was conducted to confirm the measurement construct of science efficacy (see supplementary materials). One-factor structure of science efficacy was established. Next, longitudinal measurement invariance was used to check the measurement invariance (MI) of science efficacy across three time points, T1, T3 and T4 (Table S4 in supplementary materials). There are different levels of MI, for example, configural MI (e.g., factor structure invariance, free all factor loadings and intercepts); metric MI (e.g., factor loading invariance, free all intercepts) and scalar MI (e.g., intercepts invariance). The results supported scalar MI of science efficacy (see supplementary materials Table S4), which is enough for interpreting coefficients (Chen, 2007). STEM identity and scientist career interest were each assessed by a single item, and thus the longitudinal MI cannot be established.

SEM with the latent factor of science efficacy was used to explore the longitudinal association between science efficacy, STEM identity and scientist career interests in our sample (Fig. 1). Mediation analysis explored the indirect effects of science efficacy and STEM identity on scientist career interests at T4. Specifically, two sets of mediation models were explored: the mediation pathways from science efficacy at T1 to scientist career interest at T4, and the mediation pathways from variables at T2 (i.e., science efficacy and STEM identity) to scientist career interest at T4. To control for the impact of the demographic variables, country, ethnicity and age were added to the model as control variables. Specifically, age was used to predict science efficacy at T1 and STEM identity and scientist career interest at T2. Age was controlled only for the first year. Country and ethnicity were added in the model across all time points and predicted all the variables. For simplicity, given the number of ethnic groups within our sample and allowing for the fact that the White ethnic group is the majority group in both the U.K. and U.S., as has been used in previous research (e.g., Zhao et al., 2023), the sample was split into two ethnic categories: participants identifying as White and participants identifying as non-White. A Chi-square difference test using the Satorra-Bentler scaling correction was conducted to further test the bidirectional association to investigate which direction has a significantly bigger coefficient. Using the Satorra-Bentler scaling correction is critical as indicated in the previous research for Mplus using MLR estimator (Satorra & Bentler, 2010).

The model fits for CFA and SEM were evaluated based on a joint consideration of the value of chi-square/degree of freedom (χ2/df, ≤5), the values of root mean square error of approximation (RMSEA, ≤0.08), standardized root mean square residual (SRMR, ≤0.06), comparative fit index (CFI, ≥0.90) and Tucker-Lewis index (TLI, ≥0.90) following standard recommendations (Hu & Bentler, 1999).

The intraclass correlations (ICCs) were calculated to investigate the effect of different programs (for results, see the supplementary materials). The results suggested that ICCs for scientist career interests at T2, T3 and T4 were above 0.10 (0.10, 0.12, 0.24 respectively), and STEM identity at T1 was 0.21. These findings indicated differences in STEM identity and scientist career interest between different programs. Further data analyses were conducted to understand the effect of different programs upon the model.

Adding programs as dummy-coded control variables into the model created model identification problems, which may be due to too many control variables with a limited sample size. Therefore, the largest ICCs were examined, and the model converged when only adding controls for programs on scientist career interest. The findings can be seen in the supplementary materials (Fig. S1). The majority of findings were consistent with the main findings, except that science efficacy at T1 was not significantly associated with science efficacy at T3, although the p-value was 0.051. There were not any priori hypotheses related to the differences between programs, and researchers suggest not including control variables which lack theoretical justification (Carlson & Wu, 2012). Thus, the decision to report the analyses without the control variable of programs was made.

## Results

The correlation matrix can be found in Table 1. Variables at T4 were correlated with ethnicity, which means being from a minority ethnicity group was related to lower levels of science efficacy, STEM identity and scientist career interest at T4, but all other variables from T1 to T3 were not correlated with ethnicity. Age was not corelated with any key variables in the study.

**Table 1:** Mean, Standard Deviations, and Correlations for Study Variables

	M(SD)	2	3	4	5	6	7	8	9	Ethnicity	Age
1 Science efficacy(T1)	29.73(4.09)	0.34^***^	0.21^*^	0.28^***^	0.23^**^	0.24^***^	0.21^**^	0.22^*^	0.29^***^	−0.09	0.03
2 Science efficacy(T3)	29.53(3.97)		0.42^***^	0.38^***^	0.35^***^	0.43^***^	0.19^*^	0.20^*^	0.44^**^	−0.06	−0.01
3 Science efficacy(T4)	28.77(4.29)			0.23^*^	0.36^***^	0.34^***^	0.21^*^	0.30^***^	0.37^***^	−0.25^**^	−0.09
4 STEM identity(T2)	5.23(1.75)				0.52^***^	0.51^***^	0.29^***^	0.23^*^	0.37^***^	−0.11	−0.08
5 STEM identity(T3)	5.28(1.73)					0.53^***^	0.07	0.22^*^	0.07	−0.03	−0.12
6 STEM identity(T4)	5.09(1.83)						0.08	0.25^*^	0.38^***^	−0.23^**^	−0.06
7 Scientist career interest(T2)	4.56(1.64)							0.51^***^	0.46^***^	−0.04	0.12
8 Scientist career interest(T3)	4.80(1.77)								0.62^***^	−0.14	0.05
9 Scientist career interest(T4)	4.58(2.04)									−0.28^**^	0.07

For ethnicity, white was coded as “0”, and nonwhite was coded as “1”

^*^p < 0.05; ^**^p < 0.01, ^***^p < 0.001

**Fig. 2:**
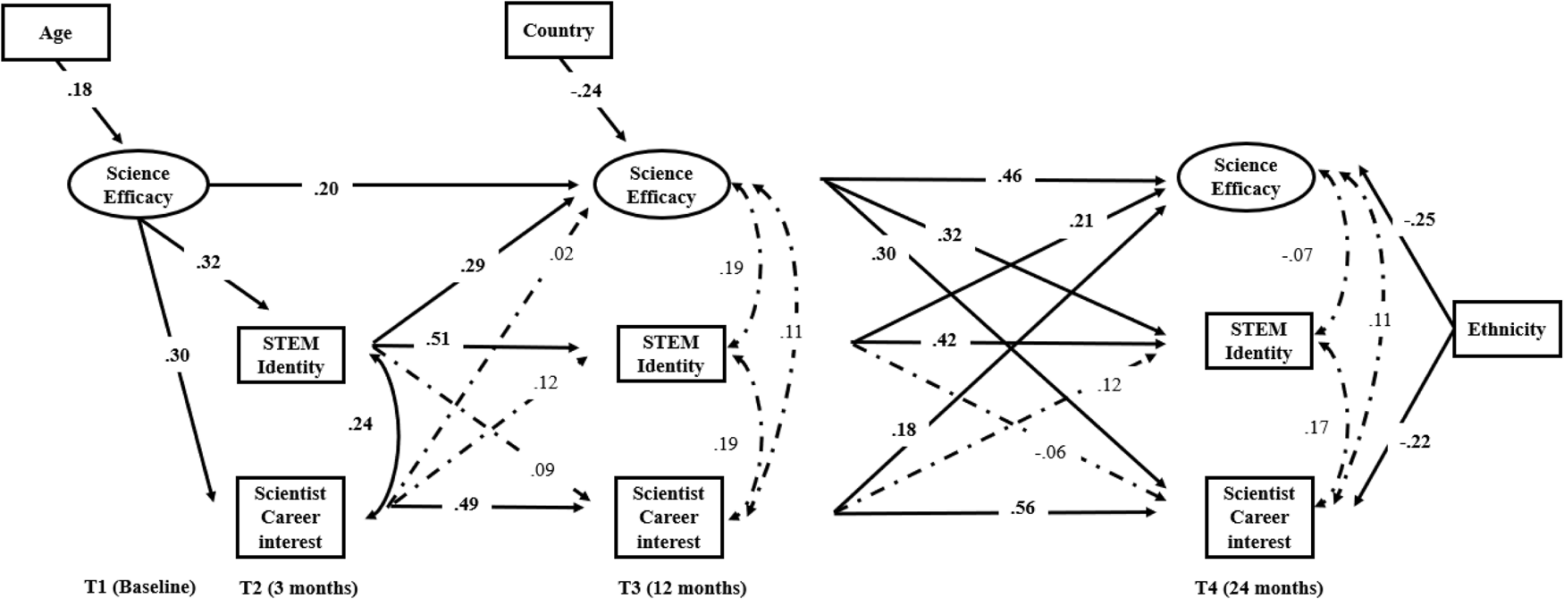
Results of Longitudinal SEM. *Note*. Bold numbers are coefficients on significant paths, and the dash lines were non-significant paths (coefficients were not bold). To simplified model, the non-significant paths related to control variables were not included in the figure

The SEM model fit the data well (Fig. 2): *χ*^*2*^(209) = 272.13, *p* = 0.002, CFI = 0.96, TLI = 0.95, RMSEA = 0.03, 95% C.I. [0.02, 04], SRMR = 0.06. For the cross-sectional association, as demonstrated in Fig. 2, high STEM identity at T2 was associated with a high level of scientist career interest at T2 (*β* = 0.24, *p* = 0.004), but there are no other significant cross-sectional associations observed.

As for the stability of the variables, science efficacy showed moderate stability across T1, T3 and T4 (*β* = 0.20/0.46, *p* = 0.03/ < 0.001, T1 to T3/ T3 to T4), and STEM identity showed moderate stability across T2, T3 and T4 (*β* = 0.51/0.42, *p* = <0.001/0.006, T2 to T3/ T3 to T4). Similarly, scientist career interests were moderately stable across all time points (*β* = 0.49/0.56, *p* = <0.001/ < 0.001, T2 to T3/ T3 to T4).

A high level of science efficacy at T1 and T3 was longitudinally associated with a high level of scientist career interest at T2 (*β* = 0.30, *p* < 0.001) and T4 (*β* = 0.30, *p* = 0.003), respectively. Only a high level of scientist career interest at T3 was linked with science efficacy at T4 (*β* = 0.18, *p* = 0.04). STEM identity did not have a direct impact on scientist career interest across time points. However, a high level of STEM identity at T2 was significantly linked with a high level of science efficacy at T3 (*β* = 0.29, *p* = 0.006). A high level of STEM identity at T3 was significantly linked with a high level of science efficacy at T4 (*β* = 0.21, *p* = 0.02). Similarly, a high level of science efficacy at T1 and T3 was linked with a high level of STEM identity at T2 (*β* = 0.32, *p* < 0.001) and T4 (*β* = 0.30, *p* < 0.001), respectively. Therefore, bidirectional associations between science efficacy and STEM identity were observed.

Chi-square difference tests were conducted to investigate if there were significant differences in the coefficients of the bidirectional paths between science efficacy and scientist career interest, as well as science efficacy and STEM identity (Table 2). The coefficient of the path from science efficacy to scientist career interest was significantly larger than the coefficient of the path from scientist career interest to science efficacy from T3 to T4. Similarly, the coefficient of the path from science efficacy to STEM identity was significantly larger than the coefficient of the path from STEM identity to science efficacy from T3 to T4.

**Table 2:** Chi-Square Test Results

Model	χ2	df	c	Comparison	Scaled χ2	Δdf	*p*
M_0_	272.13	209	1.0434				
M_1_	279.59	210	1.0441	M_1_ v.s. M_0_	6.70	1	0.010
M_2_	279.93	210	1.0432	M_2_ v.s. M_0_	8.07	1	0.005

Note. M_0_= The model without any constrains

M_1_= The model constrained the two paths of efficacy and scientist career interest equal from T3 to T4

M_2_ = The model constrained the two paths of efficacy and STEM identity equal from T3 to T4

c indicated scaling correction factor for MLR

Scaled χ2 indicated Satorra-Bentler scaled Chi Square

To examine how science efficacy and STEM identity influence scientist career interest in youth, mediation analysis was conducted to explore potential indirect effects. For scientist career interest at T4, the pathway from STEM Identity at T2 to career interest at T4 via science efficacy at T3 was significant (*β* = 0.086, SE = 0.040, *p* = 0.033). However, the pathway from science efficacy at T1 to scientist career interest at T4 via STEM identity at T2 and science efficacy at T3 was not significant.

## Discussion

Women are underrepresented in science academic pathways and careers and typically display a decline in scientist career interest during adolescence. It is critical to understand the potential pathways towards scientist career interest among girls in order to strengthen women’s likelihood of entering a science career. This study, for the first time, presents an examination of the longitudinal associations between science efficacy, STEM identity and scientist career interest among girls who participated in youth programs within informal science learning sites (science museums/centers, zoos and aquariums) in the U.K. and U.S. over a period of two years. The results highlight the importance of science efficacy in girls’ scientist career interest because science efficacy had a direct association with scientist career interest over two years. Despite no direct association observed between STEM identity and scientist career interest, the longitudinal pathway from STEM identity to scientist career interest via science efficacy was supported. Last, from 12 months (T3) to 24 months (T4), bidirectional associations between science efficacy, STEM identity and scientist career interest were observed. Specifically, T3 science efficacy predicted T4 STEM identity and T4 scientist career interest more strongly than T3 STEM identity and T3 scientist career interest predicted T4 science efficacy, and the association between science efficacy and STEM identity between T3 and T4 was bidirectional. Key to this study is evidence of reciprocal associations between science efficacy, STEM identity and scientist career interest. These findings extend previous theory and research by highlighting how these factors reinforce one another over time for girls.

Consistent with Social Cognitive Career Theory (Lent et al., 2000), science efficacy was longitudinally associated with scientist career interest over two years. Social Cognitive Career Theory (Lent et al., 2000) highlights that learning experiences and social environments are very important for an individual’s career development as they help to bolster belief in one’s ability to do work related to the career the individual would like to pursue. This fits with previous empirical findings (Sahin et al., 2017) that girls in high school who have a higher level of science efficacy were more likely to choose a STEM relevant major for university compared to those who have a lower level of science efficacy, and this suggested that among girls, science efficacy is critical for having a scientist career interest.

Social Identity Theory (Tajfel & Turner, 1986) states that identifying with a social category (e.g., STEM), namely perceiving psychological compatibility between yourself and that category, is critical for developing positive psychological outcomes. As such, girls who have a high level of STEM identity would be expected to develop science efficacy. Although previous research (Kang et al., 2019) and theory have highlighted the role of identity in developing career interest (Graham et al., 2013; Tajfel & Turner, 1986), no direct association between STEM identity and scientist career interest was observed in the current study. This may be explained by the sample characteristics and the measure of identity used in the study.

First, the sample in the current study was biased by participants with high motivation to learn STEM and high scientist career interest, because participation within informal youth programs is based upon self-selection, which is different from formal school settings when participation is compulsory. The girls in the sample may have had higher motivation and interests in STEM than participants from schools, and they may have already developed high levels of STEM identity. Second, STEM identity was assessed in the model rather than science identity. It may be possible that science identity plays a more important role than STEM identity in scientist career interest. It is appropriate to assess STEM identity in the current study given that the activities provided by programs covered broad STEM areas rather than focusing on specifically in science. The role of STEM identity in the development of scientist career interest was evident in the mediation pathway, with scientist career interest from STEM identity via science efficacy. This highlights the importance of STEM identity in scientist career interest, in line with the premise of Social Identity Theory (Tajfel & Turner, 1986) that having a shared STEM identity should facilitate the development of science efficacy.

The findings suggest that science efficacy, STEM identity and scientist career interest are mutually longitudinally related, indicating that the development of science career interest is a dynamic process, which is a new contribution to the field. Specifically, the reciprocal associations between science efficacy and STEM identity were observed, and these reciprocal associations partially support the Persistence Framework (Graham et al., 2013) which would expect that STEM identity, STEM efficacy and STEM persistence are interactive with each other. The current study extends this framework in a diverse sample of adolescent girls within the informal science learning context. The findings suggest that girls’ STEM identity can enhance science efficacy, which can then influence STEM identity. It should be noted that there is a limitation of the current study, namely the study is missing an assessment of science efficacy at T2 and of STEM identity and scientist career interest at T1, and this limited the ability to further investigate the bidirectional relationships during the first three months.

The association between science efficacy and scientist career interest is reciprocal, as indicated by the bidirectional association from T3 to T4. This finding is consistent with a previous study finding (Nauta et al., 2002) that efficacy and career interests were bidirectional among college students. All the youth programs in the current study have a focus on youth career development, and this may help girls to have increased scientist career interest. Having a scientist career interest then may influence girls’ belief about their ability in science. However, this bidirectional association was not observed in the first year, and this may indicate that the effect of scientist career interest on science efficacy needs time to accrue.

As mentioned above, sample bias may have played a role in the findings of the association between STEM identity and scientist career interest. The current study focused on female participants only, as there were very few male participants in the programs. While the small sample size for males limits the ability to include males in the study, it would be interesting for future research to compare male and female participants using multigroup analysis. This analysis would shed light on any potential gender differences between the pathway coefficients as previous research has suggested gender differences in the association between efficacy and career interest (Huang et al., 2019).

Previous research has also found ethnic differences in science efficacy (Andersen & Ward, 2014), STEM identity (Hazari et al., 2013), and scientist career interest (Wang & Degol, 2013). The current study did not look at the group differences based on ethnicity within the proposed model because of our limited sample size, which meant any multiple group analysis would have lacked statistical power. Instead, ethnicity was controlled in the model. When controlling ethnicity, the sample was divided into two categories (i.e., white and non-white) following previous research (Zhao et al., 2023). It is worth noting that many of the ethnicities in the “non-white” grouping (e.g., Asian vs Black vs Hispanic/Latinx) have substantially different sociocultural backgrounds including those that pertain to STEM stereotypes and trajectories (McGee, 2018). Future studies should explore ethnic differences in these associations.

Due to the design of the study, there was a three-month gap between the assessment of science efficacy at baseline and T1 STEM identity and T1 scientist career interest, but the decision to regress T1 variables on baseline science efficacy was made given that youth may change significantly after joining the program. Although the study is not a fully cross-lagged panel design, the auto-regression effect was still controlled. Further research should use a fully cross-lagged panel design to further confirm the reciprocal associations observed in this study.

STEM may have different meanings for different people. The measure used in the current study was adopted by previous research assessing STEM identity via rating the compatibility between oneself and STEM professionals (McDonald et al., 2019) or STEM majors (London et al., 2011). Future research could think of using a more specific term(s), relevant to school youth, such as studying in STEM or doing STEM instead of using the general term STEM. A single-item measure was used to assess STEM identity and scientist career interest. Although the single-item STEM identity has been shown to be a valid and reliable measure in previous research (McDonald et al., 2019), future studies may consider multi-item measures for assessing science career interests.

All the programs included opportunities to engage with science, but they each touched on broader STEM constructs beyond just science. Two of our measures focused narrowly on science, while one addressed STEM more broadly. Future research might specifically ask for information about science, technology, engineering, and math in order to more completely understand the relationships between these constructs over time. The identity measure focused on the broader concept of STEM, but the science efficacy and scientist career interest measures only focused on science to reduce the participants’ assessment burden and to ensure that the items were easy to read and understand. Future research is encouraged to capture all dimensions of STEM efficacy and STEM career interest. The definition and operationalization of science career as choosing a career as a scientist may be too narrow. There are a variety of jobs and careers that utilize science and/or the scientific method but which may not be explicitly labelled as jobs for “scientists”. Future research could assess career interests in STEM areas more broadly to capture a wider range of career interests.

There are other variables which were not included in the model, and future research should consider these. For example, there are no specific time limits for participants to be involved in the program, but the length of time involved in the program may be useful to include as a control variable in future research. This is because with more exposure to informal science learning environments, youth may be likely to have a more positive STEM career development. Participants were recruited from six programs, and supplemental analyses indicate generally the same pattern of findings, even when controlling for programs (see supplementary materials). Further research that includes a wider range of programs might explore potential differences caused by different types of programs.

Socioeconomic status, as one of the important indicators to describe underrepresentation in STEM, was not controlled in the model as the relevant data was not collected. Previous research suggested the invalidity and missingness of socioeconomic status data reported by adolescents (Wardle et al., 2002). With the development of approaches to measuring socioeconomic status, future research may consider subjective socioeconomic status reported by adolescents (Goodman et al., 2001), or invite parents to report socioeconomic status, to understand the effect of socioeconomic status in the psychological process of STEM development.

This research extends and synthesizes Social Cognitive Career Theory (Lent et al., 2000) with Social Identity Theory (Tajfel & Turner, 1986) and the Persistence Framework (Graham et al., 2013), and one of the key findings of the current research is the reciprocal associations between science efficacy, STEM identity and science career interest among adolescent girls. The results suggest that it is crucial to consider these key psychological concepts when understanding the underrepresentation of females in STEM and how to increase their engagement. Research suggests that educational programs can enhance self-efficacy with impacts on youth’s career development and aspirations (Falco & Summers, 2019). STEM youth programs within informal science learning sites should consider establishing activities and an inclusive environment which enhance girls’ science efficacy, and sense of STEM identity. Research reveals some specific real-life activities that could increase STEM identity and efficacy, such as interacting with role models, and having mentors and peers (Gladstone & Cimpian, 2021; Steinke et al., 2022). Those may be useful to be considered in informal science learning contexts.

Career-related experience is also valuable because the reciprocal findings suggest that scientist career interest can also enhance science efficacy. This research documents longitudinal pathways of scientist career interest among girls participating in STEM youth programs, and this further supports the importance of STEM youth programs within informal science learning contexts. STEM youth programs can serve as an important intervention to inhibit the decline in science career interests among girls, by promoting their STEM identity during adolescence and increasing STEM representation for females down the line. Therefore, policies at state, national and international levels should support informal learning sites and STEM youth programs in creating a welcoming learning community for those underrepresented in STEM.

## Conclusion

Previous theory and research have emphasized the importance of efficacy and identity in the development of career interest but have not examined longitudinal pathways involving science efficacy, STEM identity and scientist career interest. This study did this within an informal science learning context among adolescent girls who participated in STEM youth programs. The current study underscores that a higher level of science efficacy was longitudinally associated with a higher level of STEM identity and scientist career interest. Despite no direct association between STEM identity and scientist career interest, the study found for the first time a longitudinal pathway from STEM identity to scientist career interest via science efficacy. Among adolescents participating in youth programs at informal science learning sites, science efficacy and STEM identity mutually influence each other. This finding suggests that enhancing their science efficacy and STEM identity is key to promoting adolescents’ interest in being a scientist. These results have important implications for educators and informal science practitioners, especially to cultivate practices and policies which facilitate the development of science efficacy and STEM identity among adolescents.

### Supplementary information

Supplementary Information
