# Supplementary material for: Reciprocal Associations Between Science Efficacy, STEM Identity and Scientist Career Interest Among Adolescent Girls within the Context of Informal Science Learning
Source: J Youth Adolesc. 2023 Oct 11;53(2):472–84. doi: 10.1007/s10964-023-01868-6 (PMC10764556; doi:10.1007/s10964-023-01868-6)
Supplement: Supplementary file 1 — Supplementary Information [file 10964_2023_1868_MOESM1_ESM.docx]

**Supplementary Materials For Reciprocal Associations Between Science Efficacy, STEM Identity And Scientist Career Interest Among Adolescent Girls Within The Context Of Informal Science Learning**

Mengya Zhao, Emine Ozturk, Fidelia Law, Angelina Joy, Ashley R. Deutsch, Christina S. Marlow, Channing J. Mathews, Luke McGuire, Adam J. Hoffman, Frances Balkwill, Karen P. Burns, Laurence Butler, Marc Drews, Grace Fields, Hannah Smith, Mark Winterbottom, Kelly Lynn Mulvey, Adam Hartstone-Rose, Adam Rutland

**Content**

Table S1 CFA results of science efficacy (page 2)

Table S2 Factor loading of science efficacy (page 2)

Table S3 Descriptive analysis of items and missing data description (page 3)

Table S4 longitudinal measurement invariance of science efficacy (page 4)

Items used in the study (page 4-5)

Intraclass correlation coefficients (page 5)

Figure S1. SEM results controlling sites (page 6)

Table S1 CFA results of science efficacy

| Model | *χ^2^* | *df* | *p* | CFI | TLI | RMSEA[C.I.] | SRMR |
| --- | --- | --- | --- | --- | --- | --- | --- |
| Model_T1 (N=279) | 24.83 | 5 | <.001 | .965 | .930 | .119[.075, .168] | .035 |
| Model_T1_1(N=279) | 1.19 | 4 | .88 | 1.00 | 1.00 | <.001[<.001, .044] | .006 |
| Model_T3(N=162) | 4.43 | 5 | .49 | 1.00 | 1.00 | <.001[<.001, .103] | .019 |
| Model_T4(N=158) | 5.03 | 5 | .41 | 1.00 | 1.00 | <.001[<.001, .111] | .017 |

Note. Model_T1_1 is modified model with two item (Item 4 and item 5) residual correlated in order to improve the RMSEA for Model_T1

Table S2 Factor loading of science efficacy

| Model | Item 1 | Item 2 | Item 3 | Item 4 | Item 5 |
| --- | --- | --- | --- | --- | --- |
| Model_T1 | .88 | .79 | .88 | .72 | .73 |
| Model_T1_1 | .89 | .80 | .89 | .69 | .70 |
| Model_T3 | .87 | .75 | .83 | .75 | .79 |
| Model_T4 | .81 | .73 | .87 | .74 | .75 |

Note. Item 4 with Item 5 correlation was .36

Table S3 Descriptive analysis of items and missing data description

| Variable | n | number of missingness | Mean | SD | Min | Max |
| --- | --- | --- | --- | --- | --- | --- |
| SE1_T1 | 279 | 29 | 6.08 | .92 | 2 | 7 |
| SE2_T1 | 278 | 30 | 5.44 | .85 | 3 | 7 |
| SE3_T1 | 279 | 29 | 5.93 | 1.11 | 2 | 7 |
| SE4_T1 | 276 | 32 | 6.23 | .96 | 2 | 7 |
| SE5_T1 | 278 | 30 | 6.06 | 1.03 | 2 | 7 |
| SCI_T2 | 177 | 131 | 4.56 | 1.64 | 1 | 7 |
| SI_T2 | 151 | 157 | 5.23 | 1.75 | 1 | 7 |
| SE1_T3 | 162 | 146 | 6.04 | .88 | 1 | 7 |
| SE2_T3 | 162 | 146 | 5.48 | .85 | 2 | 7 |
| SE3_T3 | 162 | 146 | 5.95 | .98 | 2 | 7 |
| SE4_T3 | 161 | 147 | 6.10 | .98 | 1 | 7 |
| SE5_T3 | 162 | 146 | 5.96 | 1.02 | 2 | 7 |
| SCI_T3 | 159 | 149 | 4.80 | 1.77 | 1 | 7 |
| SI_T3 | 138 | 170 | 5.28 | 1.73 | 1 | 7 |
| SE1_T4 | 158 | 150 | 5.89 | .96 | 2 | 7 |
| SE2_T4 | 158 | 150 | 5.41 | .88 | 2 | 7 |
| SE3_T4 | 158 | 150 | 5.72 | 1.31 | 1 | 7 |
| SE4_T4 | 156 | 152 | 5.88 | 1.00 | 2 | 7 |
| SE5_T4 | 158 | 150 | 5.89 | 1.01 | 2 | 7 |
| SCI_T4 | 146 | 162 | 4.58 | 2.04 | 1 | 7 |
| SI_T4 | 127 | 181 | 5.09 | 1.83 | 1 | 7 |

Note. SE= Science efficacy; SI = STEM identity; SCI = science career interest

Table S4 Longitudinal measurement invariance of science efficacy (N=294)

| Model | *χ^2^* | *df* | *p* | CFI | TLI | RMSEA[C.I.] | Model comparison | ΔCFI | ΔRMSEA | Decision |
| --- | --- | --- | --- | --- | --- | --- | --- | --- | --- | --- |
| Model1 | 73.82 | 71 | .39 | .998 | .997 | .012[<.001, .036] |  |  |  |  |
| Model2 | 85.21 | 79 | .30 | .996 | .994 | .016[<.001, .038] | Model 1 vs Model 2 | .002 | -.004 | Accept |
| Model3 | 99.13 | 87 | .18 | .992 | .990 | .022[<.001, .040] | Model 2 vs Model 3 | .004 | -.006 | Accept |

Note. Model 1= Configural measurement invariance.

Model 2 = Metric measurement invariance.

Model 3= Scalar measurement invariance.

**Items used in the study**

**Science efficacy**

1. How good are you at science?

Not at all good/Not good/A little not good/Unsure/A little good/Good/Very good

1. If you were to list all the students from best to worst in science, where are you?

Not at all good/Not good/A little not good/Unsure/A little good/Good/Very good

1. Compared to other subjects, how good are you at science?

Not at all good/Not good/A little not good/Unsure/A little good/Good/Very good

1. How well do you expect to do in science next year?

Not well at all/Not well/A little not well/ Unsure/ A little well/ Well/ Very well

1. How good would you be at learning something new in science?

Not at all good/Not good/A little not good/Unsure/A little good/Good/Very good

**STEM identity**

Please look carefully at these pictures and then answer the question below. Select one of the 7 pairs of overlapping circles shown below that best represents how compatible you think your two identities are.

You and STEM


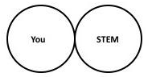

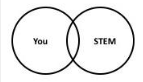

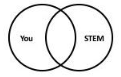

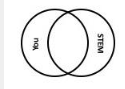

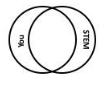

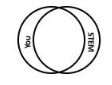


**Scientist career interest**

Do you think that you will be a scientist when you choose a career?

Absolutely not/No/Not really/Unsure/Kind of/ Yes/ Absolutely

**Intraclass correlation coefficients**

|  | **T1** | **T2** | **T3** | **T4** |
| --- | --- | --- | --- | --- |
| **Science efficacy** |  |  |  |  |
| **Item 1** | .086 | -- | .072 | .025 |
| **Item 2** | .037 | -- | .074 | .016 |
| **Item 3** | .103 | -- | .287 | .028 |
| **Item 4** | .138 | -- | .055 | .068 |
| **Item 5** | .038 | -- | .076 | .017 |
| **STEM identity** | -- | .056 | .032 | .206 |
| **Scientist career interest** | -- | .095 | .118 | .235 |


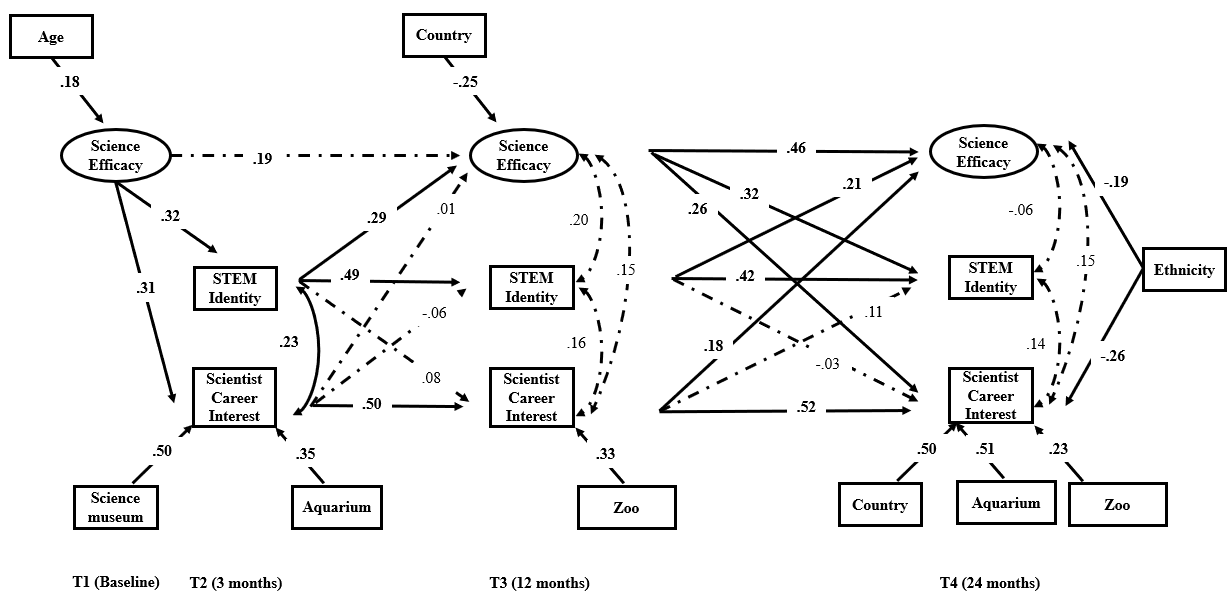


**Figure S1. SEM results controlling sites (N=285)**

**Note.** (1)The model fits were acceptable: χ2(283) = 484.49, CFI =.937, TLI =.920, RMSEA =.039, 90% C.I. [.030, 047], SRMR=.067;(2) the pathway from T2 STEM identity to T4 scientist career interest via T3 science efficacy was significant (β = .076, SE=.038, p = .045), and the pathway from T1 science efficacy to T4 scientist career interest via T2 STEM identity and T3 science efficacy was not significant.

(3) We created four dummy variables to represent a. science museum, b. aquarium, c. zoo, d. medical science education centre, e. combined children’s museum and medical heritage museum. The variable representing medical science education centre was not associated with scientist career interest at any time points.
